# Global and local impacts of multi-dimensional urban form on PM2.5 concentrations: A case study of the downstream urban agglomeration in the Yangtze River Delta

**DOI:** 10.1371/journal.pone.0354380

**Published:** 2026-07-24

**Authors:** Peng Tang, Xiaodong Yang, Xuxue Sun, Xiandi Zheng

**Affiliations:** School of Environmental Ecology, Jiangsu Open University, Nanjing, China; Zhejiang Agriculture and Forestry University: Zhejiang A and F University, CHINA

## Abstract

The acceleration of urbanization and the expansion of population scale have led to increasingly prominent PM2.5 pollution. Taking the urban agglomeration in the lower reaches of the Yangtze River within the Yangtze River Delta as the study area, this research innovatively constructs a 1D-2D-3D multi-dimensional urban form indicator system. By integrating spatial autocorrelation analysis, hot spot analysis, the Optimal parameters-based geographical detector (OPGD) model, and the Geographically Weighted eXtreme gradient boosting (GW-XGBoost) model, this study systematically reveals the spatiotemporal evolution characteristics of PM2.5 concentration from 2014 to 2022 and the global and local driving mechanisms of multi-dimensional urban form on PM2.5 concentration. This study aims to fill existing research gaps and provide scientific support for the precise prevention and control of PM2.5 pollution in urban agglomerations. The results indicate that: Multi-dimensional urban form exhibits significant spatial differentiation, with high values of 1D and 2D forms concentrated east of Nanjing, while high values of 3D forms are distributed west of Nanjing. The correlation coefficients of Building density (BD) with Mean building height (MBH) and Floor area ratio (FAR) reach 0.78 and 0.85, respectively, indicating a distinct characteristic of urban vertical expansion. The regional annual average PM2.5 concentration shows a continuous downward trend (decreasing from 60.77 μg/m^3^ in 2014 to 29.52 μg/m^3^ in 2022), with reductions ranging from 29.55% to 40.61% across individual cities. Spatially, a hot spot area at the 99% confidence level, centered around Nanjing and Ma’anshan, is formed, presenting a stable pattern of “high in the middle and low on both sides.” Regarding the global driving mechanisms, River density (RD), Digital elevation model (DEM), and Road network density (RND) are the core factors influencing PM2.5 concentration (with *q*-values of 0.3232, 0.2604, and 0.1852, respectively). Pollution risk is highest when DEM is in the 19–34 m elevation zone (concentration reaching 30.06 μg/m^3^), and all factor interactions exhibit nonlinear enhancement effects. Significant spatial non-stationarity exists in the local driving mechanisms. The regulatory effects of urban form are stronger in core cities (Nanjing, Shanghai), with local R^2^ values ranging from 0.30 to 0.35. Specifically, RD exhibits a significant positive driving effect in the central region of Nanjing–Ma’anshan–Wuhu (coefficient 0.80–1.00). In key transportation areas such as northern Shanghai, the coefficient of RND reaches 0.50–0.70. The positive effect of Proportion of transportation land (PTL) is prominent along expressways and around logistics hubs (coefficient 0.30–0.50). In contrast, in peripheral cities (Anqing, Chizhou), the local coefficients of determination (R^2^) are only 0.16–0.20. The mitigating negative effect of the Proportion of water body (PWB) exhibits a “water-adjacent attenuation” characteristic. This study effectively compensates for the shortcomings of traditional research in the systematic integration and methodological applications of characterizing nonlinear relationships, accounting for spatial heterogeneity, and analyzing multi – dimensional urban form systems. It provides scientific support and specific pathway references for the precise prevention and control of PM2.5 pollution and urban form optimization at the urban agglomeration scale. The findings carry important practical value for air quality improvement and sustainable development in similar high – density urban agglomerations.

## 1. Introduction

PM2.5, as a common airborne particulate matter, poses a significant threat to public health in cities worldwide [1]. Its concentration reflects, to a certain extent, the level of urban air quality [2]. In recent years, numerous studies have indicated that PM2.5 poses severe risks to public health and has been confirmed to be closely associated with various chronic diseases, such as cardiovascular diseases and lung cancer [3]. The severity of air pollution is closely linked to the process of urbanization, where accelerated urbanization and population growth are typically accompanied by an increase in PM2.5 emissions [4]. Particularly in urban central areas, the high concentration of commercial, residential, and transportation land uses, along with the dense construction of high-rise buildings, results in distinct regional and seasonal characteristics in PM2.5 concentrations [5]. Meanwhile, as the green attributes of production diminish, corporate risk tends to increase [6]. Furthermore, the degree of greenness in production also leads to changes in product value, emerging as one of the key factors influencing market fluctuations [7]. Therefore, research on the influencing mechanisms of PM2.5 is of great significance, as it provides a scientific basis for air quality management and improvement.

In recent years, research on PM2.5 has primarily focused on risk assessment and the investigation of driving factors. Risk assessment employs trend analysis methods, such as the Mann-Kendall test, to calculate the temporal variations of PM2.5 [8], thereby predicting PM2.5 pollution risks. Recent studies on the driving factors of PM2.5 have covered a wide range of aspects, including policy [9], population exposure [10,11], climate and temperature [12], urban land use types [13], as well as green space coverage and NDVI. Some studies have specifically focused on residential and neighborhood scales, exploring the impacts of spatial configuration, building morphology, and three-dimensional green volume on PM2.5 concentrations [14–16].

Against the macro backdrop of economic pressure and structural adjustment, traditional industries have continuously squeezed the development space of high-tech industries [17], which directly affects regional development intensity and urban morphological structure, further alters pollution emission intensity and local environmental quality, and exerts sustained potential impacts on regional air quality. Among these, urban form is recognized as one of the significant factors influencing air quality [18], affecting PM2.5 concentrations by influencing vehicle usage, green space regulation, pollutant dispersion, and the urban heat island effect [19]. However, existing research still has limitations. First, the research scale and indicator system are restricted: most studies are limited to small scales such as neighborhoods or individual cities, and have not comprehensively considered the integrated impacts of systematic urban form indicators on PM2.5 concentrations. Second, the analysis of global and local driving mechanisms remains incomplete. Most existing studies adopt traditional regression model methods [20] or machine learning approaches. Traditional regression models cannot capture the complex nonlinear relationships between PM2.5 and its driving factors. While some studies employ machine learning methods such as Random forest (RF), Gradient boosting decision trees (GBDT), and eXtreme gradient boosting (XGBoost) [21,22] to analyze the nonlinear driving mechanisms of PM2.5 concentrations, both approaches inherently assume spatial homogeneity in the driving relationships. They fail to adequately account for the spatial non-stationarity characteristics of how different drivers influence PM2.5 (Yuan, et al. 2018). This limitation reduces the model’s explanatory power for the spatial differentiation of PM2.5, consequently affecting the accuracy and spatial applicability of the revealed driving mechanisms. Therefore, how to integrate global nonlinear detection and local heterogeneity modeling, construct a multi-dimensional urban form indicator system, and comprehensively and accurately reveal the impacts of urban form on PM2.5 concentrations at the urban agglomeration scale has become a key scientific issue urgently to be solved in current air quality and urban spatial research. It is also an important topic with practical significance that addresses existing research gaps, thereby providing solutions for optimizing air quality regulation in high-population-density regions.

Geographical detector (GD) is capable of detecting spatial heterogeneity and quantifying the impact of driving factors on the overall spatial differentiation pattern [23]. However, GD faces challenges in selecting discretization parameters [24], which limits model precision and necessitates parameter optimization to enhance its effectiveness. Moreover, hybrid models that integrate the spatial analysis framework of Geographically weighted regression (GWR) with the strengths of machine learning algorithms offer a superior technical pathway for analyzing the complex associations between the built environment and PM2.5 concentrations. These models retain the strong learning capacity of machine learning for nonlinear relationships while fully considering spatial heterogeneity through their geographically weighted properties [25]. This approach has emerged as a highly promising research method and is progressively being applied in urban planning studies [26–28]. This study innovatively employs the Optimal parameters-based geographical detector (OPGD) and Geographically weighted eXtreme gradient boosting (GW-XGBoost) model as technical tools to investigate the impacts of urban form on PM2.5 concentrations, enabling global- and local-scale impact analyses.

The downstream Yangtze River Delta region, home to megacities such as Nanjing and Shanghai, exhibits high population density and relatively poor air quality [29]. Understanding the distribution characteristics and influencing mechanisms of air pollution is crucial for revealing the underlying physical processes of regional PM2.5 and formulating effective environmental policies [30,31]. This study systematically constructs a multi-dimensional urban form indicator system encompassing 1D, 2D, and 3D dimensions. It analyzes the spatiotemporal distribution patterns of urban form and PM2.5 concentration in the lower reaches of the Yangtze River within the Yangtze River Delta. By integrating the Optimal parameters-based geographical detector (OPGD) and the Geographically weighted eXtreme gradient boosting (GW-XGBoost) model, this study aims to address the following key questions: (1) What are the characteristics of PM2.5 concentration in the lower reaches of the Yangtze River urban agglomeration within the Yangtze River Delta? (2) What are the global driving mechanisms of PM2.5 concentration? (3) What are the local driving mechanisms of PM2.5 concentration?

## 2. Study area and data sources

### 2.1. Study area overview

**Fig 1:**
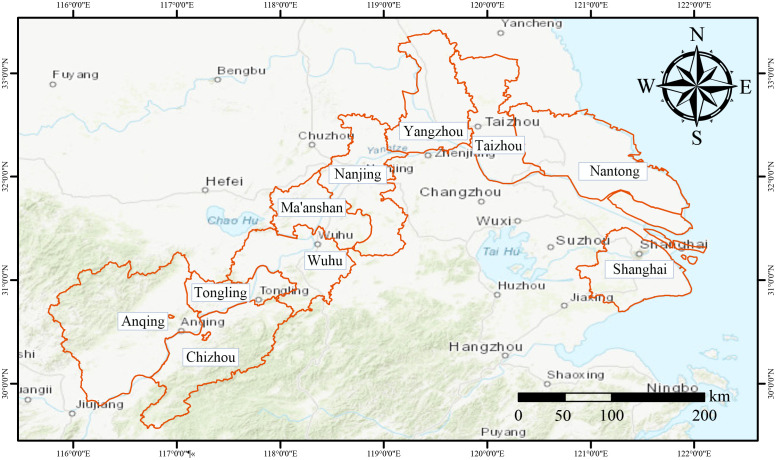
Map of the study area.

The downstream Yangtze River region encompasses cities within the provinces of Shanghai, Jiangsu, and Anhui. Within the Yangtze River Delta (YRD) region, the major cities include Anqing, Chizhou, Tongling, Wuhu, Ma’anshan, Nanjing, Yangzhou, Taizhou, Nantong, and Shanghai (see **Fig 1**). Located on the alluvial plain at the estuary of the Yangtze River, the YRD is characterized by a dense river network and low-lying topography, primarily influenced by a subtropical monsoon climate with significant seasonal weather variations [32]. As one of China’s most economically dynamic regions, the downstream Yangtze River urban areas exhibit high rates of economic growth and urbanization, coupled with dense populations, resulting in persistently high levels of PM2.5 emissions [33]. Following successive key initiatives outlined in the Five-Year Plan for air pollution control and the “Ten Measures for Air Pollution Prevention and Control” [34,35], PM2.5 concentrations in the Yangtze River Delta region have entered a relatively stable phase characterized by a decelerating rate of decline.

### 2.2. Data sources

The research data consist of two categories: PM2.5 data and urban form indicator data. The PM2.5 concentration data were sourced from the dataset shared by Haimeng Liu on the Science Data Bank platform. This dataset was constructed by first collecting globally calibrated ground-observed PM2.5 concentration data, which were processed using GWR by Washington University in St. Louis, along with data from Chinese air quality monitoring stations. Subsequently, the Zonal Statistics tool in ArcGIS software was employed to calculate the annual average PM2.5 concentrations for administrative units. Land use classification data, 3D building datasets, Digital elevation model (DEM), road network data, and river data were obtained from various open-source platforms, including the Natural Resources Bureau, Baidu Online Maps, and Geospatial Data Cloud. The reliability of these data sources is well-established (see **Table 1**).

**Table 1:** Data sources.

Data type	Temporal coverage	Spatial resolution	Source
PM2.5 concentration	2014-2022	1km	Zenodo – CHAP Dataset (https://zenodo.org/communities/chap/)
Land use classification	2022	30m	RESDC – CAS (https://www.resdc.cn/)
3D building dataset	2022	–	Baidu Map Open Platform(https://lbsyun.baidu.com/)
DEM	2022	30m	Geospatial Data Cloud(http://www.gscloud.cn/)
Road network	2022	–	National Catalogue Service for Geographic Information(https://www.webmap.cn/)
River data	2022	–	Scientific DataNational Earth System Science Data Center (http://www.geodata.cn/)

## 3. Methods

**Fig 2:**
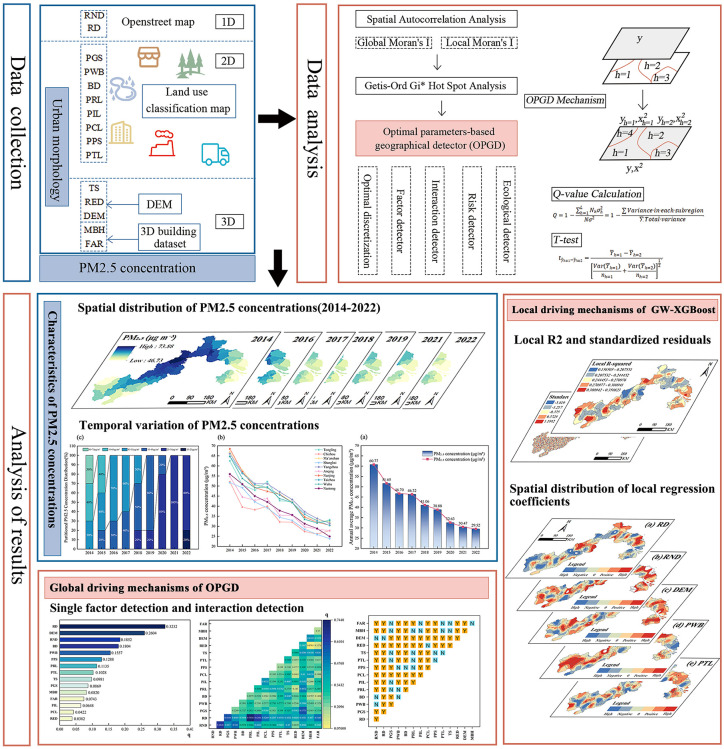
Research flowchart.

This research innovatively constructs a multi-dimensional urban form indicator system, utilizing open-source data on urban land use, buildings, and topography from platforms such as Open Street Map, Baidu Online Maps, and the Natural Resources Bureau. Integrated with spatially distributed PM2.5 concentration data obtained from the Scientific Data Bank, spatial autocorrelation analysis and hotspot analysis were conducted. Subsequently, from a global perspective, an OPGD model was developed to perform factor detection, interaction detection, risk detection, and ecological detection of PM2.5 concentrations. From a local perspective, a GW-XGBoost model was constructed to analyze the local determination coefficients of urban form factors as a whole and to quantify the local regression coefficients of core factors. Ultimately, this approach enables a dual-perspective analysis—both across multiple urban form dimensions and from global to local scales—of the driving mechanisms behind PM2.5 concentrations (see **Fig 2**).

Spatial autocorrelation analysis and hot spot analysis were conducted annually for the period 2014–2022, using the annual average PM2.5 concentration for each corresponding year. This approach aims to reveal the long-term trends and spatial pattern evolution of PM2.5 concentration. To ensure that the research findings reflect pollution characteristics under typical socio‑economic development conditions, this study adopts 2022 as the baseline year for analysis. The OPGD model, OLS regression, and GW-XGBoost model were conducted exclusively for the year 2022. All urban form predictor variables (1D-2D-3D indicators) are based on 2022 data, and the dependent variable is the PM2.5 concentration of the year 2022, ensuring temporal consistency in the analysis of driving relationships. This choice avoids the anomalous fluctuations in air quality induced by traffic restrictions and industrial shutdowns during the COVID‑19 pandemic control period of 2020–2021 [36]. Moreover, under the long‑term and continuous implementation of air‑pollution control policies [37], the marginal abatement effects of these policies have stabilised. As a result, regional pollution patterns in 2022 are less perturbed by policy interventions [38] and are more likely to be governed by long‑term structural factors such as urban form, making this year particularly suitable for robustly revealing the influence mechanisms of urban form on air pollution. This study aims to investigate the long-term and stable impacts of urban forms on PM2.5 concentrations. Given that meteorological conditions in the single year of 2022 are year-specific and volatile, they may confound the estimated effects of urban forms and weaken the universality and accuracy of the results. Therefore, they are not considered or included as moderating variables in the effect estimation.

### 3.1. Selection and correlation analysis of urban form indicators

This study constructs a multi-dimensional indicator system to characterize urban form, encompassing 1D, 2D, and 3D indicators, providing a comprehensive reflection of urban form features. Specifically, 1D indicators capture the linear structure of a city [39], 2D indicators focus on land use patterns, and 3D indicators address three-dimensional aspects such as building and terrain morphology [40] (see **Table 2**). The indicators of the three dimensions respectively characterize the features of urban form dominated by linear or network elements, those dominated by planar elements, and the three-dimensional features dominated by elevation or vertical structure, with each dimension distinguishing different urban form elements. In ArcGIS, the values of each indicator were calculated in the attribute table according to the corresponding formula, and then classified into five levels using the natural breaks (Jenks) method.

**Table 2:** Urban form indicator system.

Category	Abbreviation	Description	Calculation	Details
1D	*RND*	Density of roads	$RND=\frac{\sum_{i=\text{1}}^{n}L_{i}}{A}$	Where, $L_{i}$ is the total length of *i-th* road within the grid cell, an *A* is the total area of the study unit.
	*RD*	Density of rivers	$RND=\frac{\sum_{i=\text{1}}^{n}l_{i}}{A}$	$l_{i}$ is the length of river *i* within the grid cell.
2D	*PGS*	Percentage of green space area relative to the block area	$PGS=\frac{A_{GS}}{A}$	$A_{GS}$ is the green space area.
	*PWB*	Percentage of water body area relative to the block area	$PWB=\frac{A_{WB}}{A}$	$A_{WB}$ is the water body area.
	*BD*	Ratio of the total building floor area within a block to the block area	$BD=\frac{\sum_{i=\text{1}}^{n}A_{i}}{A}$	Where, $A_{i}$ is the total building floor area within the grid cell, and *A* is the total area of the study unit.
	*PRL*	Percentage of residential area relative to the block area	$PRL=\frac{A_{RL}}{A}$	$A_{RL}$ is the residential area.
	*PIL*	Percentage of industrial area relative to the block area	$PIL=\frac{A_{IL}}{A}$	$A_{IL}$ is the industrial area.
	*PCL*	Percentage of commercial area relative to the block area	$PCL=\frac{A_{CL}}{A}$	$A_{CL}$ is the commercial area.
	*PPS*	Percentage of public service area relative to the block area	$PPS=\frac{A_{PS}}{A}$	$A_{PS}$ is the public service area.
	*PTL*	Percentage of transportation land area relative to the block area	$PTL=\frac{A_{TL}}{A}$	$A_{TL}$ is the transportation land area.
3D	*TS*	Steepness of the land surface	$TS=\frac{S_{i}}{n}$	$S_{i}$ is the slope of the *i-th* grid cell, and *n* is the number of grid cells.
	*RED*	Difference in elevation	$RE=\frac{H_{i}-H_{min}}{H_{max}-H_{min}}$	$H_{i}$ is the mean elevation of the *i*-th block, and $H_{max}$ and $H_{min}$ represent the maximum and minimum elevation within the study area, respectively.
	*DEM*	Average elevation of the terrain within the block	$DEM=\frac{\sum_{i=\text{1}}^{n}h_{i}}{n}$	$h_{i}$ is the elevation of the *i-th* grid cell, and *n* is the number of grid cells.
	*MBH*	Average height of all buildings within the block	$MBH=\frac{\sum_{i=\text{1}}^{n}H_{i}}{n}$	$H_{i}$ is the height of the *i-th* building, and m is the number of buildings within the block.
	*FAR*	Ratio of the total building floor area within a block to the total block area	$FAR=\frac{\sum_{i=\text{1}}^{n}A_{i}F_{i}}{A}$	$A_{i}$ is the footprint area of the *i-th* building, $F_{i}$ is the number of floors of the *i-th* building, and *A* is the total area of the study unit.

Road network density (RND) and River density (RD) were selected as 1D urban form indicators. Roads support the majority of urban vehicular traffic, thereby contributing to increased PM2.5 emissions [41]. Riverbanks are often densely populated and prone to forming local thermal circulation, which can lead to the accumulation of PM2.5 within river valleys [29].

Proportion of green space (PGS), Proportion of water body (PWB), Building density (BD), Proportion of residential land (PRL), Proportion of industrial land (PIL), Proportion of commercial land (PCL), Proportion of public service area (PPS), and Proportion of transportation land (PTL) were selected as 2D urban form indicators. These metrics reflect urban land use types, which are manifestations of human activities and have a significant influence on the distribution of PM2.5 [42].

Terrain slope (TS), Relative elevation difference (RED), DEM, Mean building height (MBH), and Floor area ratio (FAR) were selected as 3D urban form indicators. TS, RED, and DEM reflect topographical relief characteristics, which not only influence meteorological conditions but may also induce atmospheric disturbances such as temperature inversions and vortices, thereby affecting the distribution of PM2.5 [43]. MBH and FAR represent the spatial volume of structures. High-rise buildings have been demonstrated to affect wind speed and airflow, and can even contribute to urban heat island effects, increasing energy consumption and consequently leading to the accumulation and elevated emissions of PM2.5 around buildings [44].

These indicators are key metrics in the urban ecological environment [45] and significantly influence changes in PM2.5 concentrations. Correlation analysis was conducted on all form indicators to investigate the strength and direction of interactions among different urban form factors.

### 3.2. Spatial autocorrelation analysis

Spatial autocorrelation analysis of PM2.5 concentration was conducted annually for the period 2014–2022, using the annual average PM2.5 concentration data for each corresponding year, to investigate the spatial agglomeration characteristics of PM2.5 concentration in each year. This analysis aims to measure the correlation and clustering patterns of PM2.5 concentrations across different spatial units.Spatial autocorrelation analysis is divided into global spatial autocorrelation and local spatial autocorrelation. The former describes the overall spatial characteristics of attribute values across the entire study area. The latter identifies the spatial association or variation between each spatial location and its neighboring areas within the study region. The association patterns can be categorized into five types: high-high, low-low, high-low, low-high, and not significant [46]. The calculation formulas are as follows:


$$\begin{array}{c} Global\;Moran^{\prime }s\;I=\frac{n\sum_{i=\text{1}}^{n}\sum_{j=\text{1}}^{n}W_{ij}\left(X_{i}-\overset{―}{X}\right)\left(X_{j}-\overset{―}{X}\right)}{\sum_{i=\text{1}}^{n}\sum_{j=\text{1}}^{n}W_{ij}\sum_{i=\text{1}}^{n}{\left(X_{i}-\overset{―}{X}\right)}^{\text{2}}} \end{array}$$ (1)

$$\begin{array}{c} Local\;Moran^{\prime }s\;I=\frac{n\left(X_{i}-\overset{―}{X}\right)}{\sum_{i=\text{1}}^{n}\left(X_{i}-\overset{―}{X}\right)}\sum_{j=\text{1},j\neq i}^{n}W_{ij}\left(X_{j}-\overset{―}{X}\right) \end{array}$$ (2)

Where, *n* represents the number of spatial units in the study area, $W_{ij}$ is the spatial weight matrix indicating the spatial relationship between units *i* and *j,* $X_{i}$, $X_{j}$ are the attribute values of units *i* and *j*, respectively, $\overset{―}{X}$ is the mean value of the attribute across all units, $\left(X_{i}-\overset{―}{X}\right)$ represents the deviation of the attribute value for unit i from the mean value.

### 3.3. Hotspot analysis

This study employs the Getis-Ord Gi* hot spot analysis method to identify the spatial clustering patterns of PM2.5 concentration from 2014 to 2022, precisely locating high-concentration clusters (hot spots) and low-concentration clusters (cold spots) along with their statistical significance [47]. This method reveals the spatial agglomeration intensity of PM2.5 concentration by quantifying the difference between local areas and the global average, thereby providing a spatial pattern foundation for subsequent driving mechanism analysis [48].

In this study, 20 km × 20 km grid cells are defined as “events,” with each cell serving as an independent analysis unit to ensure uniform spatial granularity suitable for regional-scale analysis. A “global neighborhood” setting is adopted, with the threshold distance parameter set to 0, meaning that all spatial units are considered neighbors of one another (with $W_{ij}$=1 for all *i, j*). This neighborhood definition identifies hot and cold spots based on regional relative differences rather than local spatial clustering.Specifically, the *Gi* statistic reflects the relative magnitude of PM2.5 concentrations in each unit compared to the entire study area, rather than localized high or low clustering within a small local window. This ensures hotspot results are consistent with the regional-scale analytical perspective of this study and avoids misinterpreting hotspots as small-scale localized agglomerations. The global neighborhood approach comprehensively captures the spatial associations among all units within the region, avoiding the subjectivity associated with selecting a local neighborhood threshold while maintaining consistency with the spatial scale of the subsequent OPGD and GW-XGBoost models. The analysis was implemented in R version 4.2.3 using the core package spdep (version 1.2–8), with the Gi* statistic and *Z*-values calculated using the localG() function. The core principle of the Getis-Ord Gi* statistic is to determine whether a given spatial unit belongs to a significant high-value or low-value cluster by calculating the weighted sum of PM2.5 concentrations between each spatial unit and its neighboring units. The calculation formula is as follows:


$$\begin{array}{c} G_{i}^{*}\;=\frac{\sum_{j=\text{1}}^{n}W_{ij}X_{j}}{\sum_{j=\text{1}}^{n}X_{j}} \end{array}$$ (3)

Where, Gi* is the Getis-Ord Gi* statistic of an event *i* over *n* events; *n* represents the total number of spatial units in the study area; The term, $X_{j}$, characterizes the magnitude of the variable *X* at events *j* over all *n*; $W_{ij}$ represents the elements of the spatial weight matrix, defined to characterize the spatial proximity relationship between unit *i* and unit *j*. This study adopts a binary spatial weight matrix: $W_{ij}$ = 1 if units *i* and *j* are defined as neighbors (per the global neighborhood setting described above), and $W_{ij}$ = 0 otherwise. The distribution of the Gi* statistic is normal when normality is observed in the underlying distribution of the variable *X*.

For the purpose of statistical testing, the Gi* statistic needs to be standardized to obtain the *Z*-score, with the formula as follows:


$$\begin{array}{c} Z(Gi*)=\frac{\sum_{j=\text{1}}^{n}W_{ij}X_{j}-\overset{―}{x}\sum_{j=\text{1}}^{n}W_{ij}^{\text{2}}}{s\sqrt{\frac{n\sum_{j=\text{1}}^{n}W_{ij}^{\text{2}}-\left(\sum_{j=\text{1}}^{n}W_{ij}^{\text{2}}\right)}{n-\text{1}}}} \end{array}$$ (4)

Where, $\overset{―}{x}$ is the mean PM2.5 concentration of all spatial units; *s* is the standard deviation of PM2.5 concentration of all spatial units; the meanings of the remaining parameters are consistent with those in Equation (3).

Note: Positive and negative Gi* statistic with high absolute values correspond to clusters of high and low PM2.5 concentration values, respectively. A Gi* close to zero implies a random distribution of events.

### 3.4. OPGD model

Based on the baseline year data of 2022, the OPGD spatial statistical analysis was performed to reveal the spatially non-stationary driving mechanisms of global multi-dimensional urban form indicators on PM2.5 concentration, with both predictor variables and the dependent variable based on 2022 data. This study employed the GD package in the R environment to construct an OPGD model, primarily utilizing the parameter optimization module, single-factor detection module, interaction detection module, risk detection module, and ecological detection module to analyze the relationship between PM2.5 concentrations and urban form factors. A grid unit of 20 km × 20 km was defined for detection at this scale. The package provides detailed computational outputs, generates various spatial analysis results and visualizations, while significantly enhancing computational efficiency [49,50].

The first step of the OPGD model involves determining the discretization of continuous variables. Initially, optimal discretization is selected. For each urban form variable, five common discretization methods are applied: equal interval, natural breaks, quantile, geometric interval, and standard deviation. Subsequently, the number of discretization classes (ranging from 3 to 5) for each variable is considered to evaluate the granularity of discretization. Finally, the Geographical Detector is used to calculate and compare the *q*-values for each discretization combination, thereby identifying the combination yielding the highest *q*-value. Next, single-factor detection is performed. The discretized independent variables are input into the Geographical Detector model to compute the *q*-value for each factor, conducting factor detection and comparing the explanatory power of each variable on PM2.5 concentrations. In the third step, the interaction q-values between different variables are calculated to analyze how the interactions among multiple explanatory variables influence PM2.5 concentrations. Finally, risk detection is conducted.

Quantifying the explanatory power of influencing factors using the *q*-statistic is the core component of OPGD. The calculation formula for the *q*-value is as follows:


$$\begin{array}{c} Q=\text{1}-\frac{\sum_{h=\text{1}}^{L}N_{h}\sigma_{h}^{\text{2}}}{N\sigma^{\text{2}}}=\text{1}-\frac{\sum Variance\;in\;each\;subregion}{\sum Total\;variance} \end{array}$$ (5)

Where *L* represents the number of layers after discretization of the independent variable, *h* denotes the different sublayers of the variable after stratification, *N* is the total number of samples in the study area, $N_{h}$ is the number of samples in the *h*-th sublayer. $\sigma^{\text{2}}$ represents the total variance of the attribute values of all samples in the study area, and $\sigma_{h}^{\text{2}}$ represents the within-stratum variance of the samples within the *h*-th sublayer. A *q*-value close to 1 indicates a high explanatory power of the variable for PM2.5 concentration. Conversely, a *q*-value close to 0 indicates a low explanatory power of the variable for PM2.5 concentration.

A *t*-test was performed based on the average risk within each stratum of the discretized factors to compare the significance of differences in PM2.5 concentrations. The calculation formula for the *t*-statistic is as follows:


$$\begin{array}{c} t_{{\overset{―}{y}}_{h=\text{1}}-{\overset{―}{y}}_{h=\text{2}}}=\frac{{\overset{―}{Y}}_{h=\text{1}}-{\overset{―}{Y}}_{h=\text{2}}}{{\left[\frac{Var\left({\overset{―}{Y}}_{h=\text{1}}\right)}{n_{h=\text{1}}}+\frac{Var\left({\overset{―}{Y}}_{h=\text{2}}\right)}{n_{h=\text{2}}}\right]}^{\frac{\text{1}}{\text{2}}}} \end{array}$$ (6)

Where, $t_{{\overset{―}{y}}_{h=k}-{\overset{―}{y}}_{h=k+\text{1}}}$is the statistic for testing the significant difference between two adjacent strata, ${\overset{―}{Y}}_{h=k}$ is the sample means of substratum k, respectively, $Var\left({\overset{―}{Y}}_{h=k}\right)$ denote the variances of the sample means for substratum k, $n_{h=k}$ represent the sample sizes of substratum.

To test the robustness of the results, this study additionally conducted a sensitivity analysis. On the one hand, while keeping the discretization method unchanged, the number of bins was extended to a range of 2–8 for comparison. On the other hand, with the number of bins fixed at 4 (the optimal number for core factors), cross-validation was performed using the equal frequency method and the geometric interval method in comparison with the original method, systematically analyzing the response characteristics of key q-values and core driving factors. To verify the spatial robustness of the interaction results, a comparative analysis of interaction detection was conducted at three spatial unit scales: 10 km × 10 km [51], 20 km × 20 km, and 30 km × 30 km.

### 3.5. GW-XGBoost model

Based on the baseline year data of 2022, the GW-XGBoost model was constructed to reveal the spatially non-stationary driving mechanisms of local multi-dimensional urban form indicators on PM2.5 concentration, with both predictor variables and the dependent variable based on 2022 data. This study employs GW-XGBoost to investigate the spatially heterogeneous relationships between PM2.5 concentrations and multi-dimensional urban form indicators. The GW-XGBoost model is a hybrid model that integrates the spatial analysis framework of Geographically Weighted Regression (GWR) with the advantages of the XGBoost machine learning algorithm. It leverages the nonlinear learning capability of XGBoost while accounting for spatial heterogeneity, thereby demonstrating greater potential for modeling the complex influences of the built environment [27]. In the geographically weighting step, a Gaussian kernel function was used to construct the spatial weight matrix, ensuring that the weights decay smoothly with increasing distance. This approach conforms to the “distance decay” principle in geographical space and better aligns with the spatial correlation characteristics of regional pollutant dispersion. Leave-One-Out cross validation (LOOCV) was employed to select the optimal bandwidth. The bandwidth search range was set to 5–50 km (based on the 20 km × 20 km grid cells, covering a reasonable range of spatial scales within the study area). The Residual sum of squares (RSS) of the model under different bandwidths was calculated, and the bandwidth corresponding to the minimum RSS (ultimately determined to be 25 km) was selected as the optimal bandwidth. This bandwidth ensures effective weight contributions from neighboring units while avoiding model overfitting caused by an excessively small bandwidth or the masking of spatial heterogeneity caused by an excessively large bandwidth.

Initially, spatially weighted form indicators are constructed based on the local coefficients estimated by GWR. The GWR model formula is as follows:


$$\begin{array}{c} y^{\left(i\right)}=\beta_{k}^{\left(i\right)}+\sum_{k=\text{1}}^{p}\beta_{k}^{\left(i\right)}x_{k}^{\left(i\right)}+\varepsilon^{\left(i\right)} \end{array}$$ (7)

Where, $\beta_{\text{k}}^{\left(\text{i}\right)}$ denotes the local coefficient for the k-th feature at location (${\text{u}}_{\text{i}},{\text{v}}_{\text{i}}$), ${\text{x}}_{\text{k}}^{\left(\text{i}\right)}$ corresponds to the urban form indicators, and $\epsilon^{\left(\text{i}\right)}$ is the error item.

The estimation process of local coefficients $\beta^{i}\in R^{p}$ was implemented by solving:


$$\begin{array}{c} \beta^{i}={\left(X^{T}\right)W^{\left(i\right)}X}^{-\text{1}}X^{T}W^{\left(i\right)}y \end{array}$$ (8)

Where, $X\in R^{n\times p}$ is the matrix of all feature vectors, $y\in R^{n}$is the vector of PM2.5 concentration, and $W^{i}\in R^{n\times n}$ is a spatial weight matrix constructed via a Gaussian kernel.

Based on these coefficients, the spatially weighted form indicator for each unit is calculated as:


$$\begin{array}{c} {\tilde{x}}_{i}=\sum_{j\in N(i)}\beta^{\left(j\right)}\cdot x_{j} \end{array}$$ (9)

Where, N(i) denotes the neighboring blocks for block *i.*

Subsequently, the spatially weighted form indicators are directly incorporated into the training process of the XGBoost algorithm (He et al., 2024), ensuring that urban parcels with similar spatial conditions are prioritized during model fitting. Grid search combined with spatially stratified five-fold cross-validation was used for XGBoost hyperparameter optimization. Spatially stratified five-fold cross-validation performs stratified sampling based on the spatial clustering characteristics of PM2.5 concentrations, ensuring that each fold contains the complete range of spatial pattern types and avoiding validation biases associated with traditional random cross-validation caused by spatial autocorrelation. The calculation formula for the GW-XGBoost regression model is as follows [52]:


$$\begin{array}{c} \overset{ˇ}{y_{i}}=\sum_{m=\text{1}}^{M}f_{m}\left(\tilde{x_{i}}\right),f_{m}\in F \end{array}$$ (10)

where *M* is the number of decision trees, $f_{m}$ represents the *m*-th tree, and *F* denotes the space of possible regression trees.

Finally, multiple methods were employed to test the risk of model overfitting. The training and validation sets were divided using a 7:3 spatially stratified sampling ratio, and the R^2^ and RMSE of both sets were compared. The effectiveness of model complexity suppression was verified by adjusting regularization parameters. Additionally, a global *Moran’s I* test was conducted on the model residuals to verify the adequacy of spatial information extraction.

## 4. Results

### 4.1. Characteristics of multi-dimensional urban form

#### 4.1.1. Spatial distribution of multi-dimensional urban form indicators.

The differentiation in urban form reflects, to some extent, disparities in urban resources and development levels from west to east within the region [53]. The high-value areas of urban form indicators across different dimensions exhibit an east-west differentiation pattern with Nanjing as the dividing line, and the spatial distribution characteristics within each dimension vary, as specifically manifested in the following: Regarding 1D forms, high values of both RND and RD are predominantly distributed in the eastern part of the downstream Yangtze River Delta. Specifically, this includes central-northern Nanjing, the southern parts of Yangzhou, Taizhou, and Nantong, as well as most areas of Shanghai.

In terms of 2D forms, high values of the PGS, BD, PRL, PIL, and PPS are mainly located in areas east of Nanjing, exhibiting a patchy distribution along northern Nanjing and the southern parts of other cities. High values of the PWB form a belt-like pattern traversing various cities, distributed along the central parts from west to east, and shifting to the southern peripheries of cities east of Nanjing. The high-value areas of PCL and PTL are distributed along major transportation corridors in ribbon-like and patchy patterns, showing no significant spatial agglomeration.

**Fig 3:**
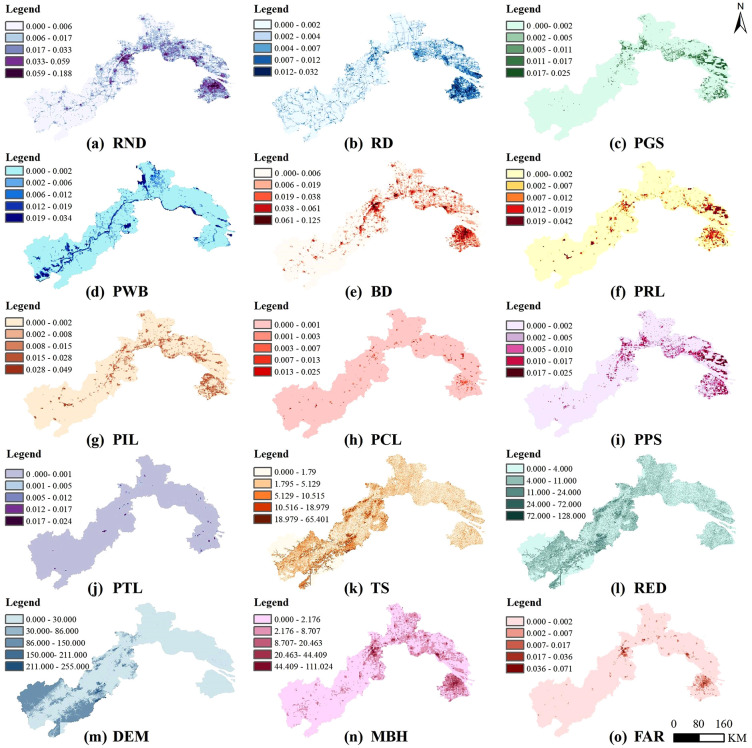
Urban form characteristics.

For 3D forms, high values of TS, RED, and DEM are primarily concentrated in areas west of Nanjing. In contrast, high values of Mean MBH and FAR are predominantly found east of Nanjing. Specifically, high TS and RED values show notable aggregation in central-northern Nanjing, northern Ma’anshan, and southern Anqing, while high DEM values are concentrated in northern Anqing and Chizhou. High MBH and FAR values are mainly clustered in central-northern Nanjing and northern Shanghai, with the high-value distributions in other eastern areas being relatively scattered (see **Fig 3**).

#### 4.1.2. Correlation analysis of multi-dimensional urban form indicators.

**Fig 4:**
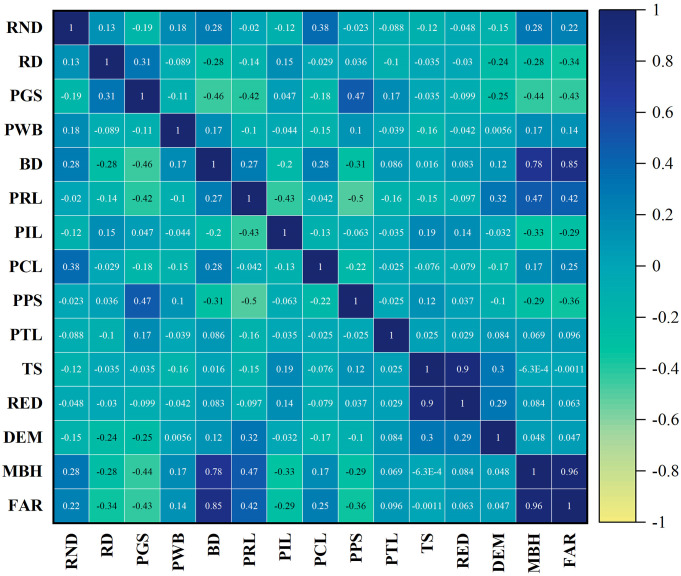
Correlation among urban form indicators.

The results of the Pearson correlation analysis indicate that the strength of positive correlations among the factors is greater than that of negative correlations. Specifically, significant positive correlations are observed between BD and MBH and FAR, with correlation coefficients of 0.78 and 0.85, respectively. Additionally, the positive correlation between FAR and MBH is extremely strong, reaching 0.96. There are relatively strong negative correlations between the PGS and BD, PRL, MBH, and FAR, with correlation coefficients of −0.46, −0.42, −0.44, and −0.43, respectively. Strong negative correlations also exist between PRL and PIL and PPS, with coefficients of −0.43 and −0.50 (see **Fig 4**).

The strong correlations among the BD, MBH, and FAR indicators reflect the functional associations between indicators across different dimensions. These three indicators collectively characterize “building development intensity” from the three dimensions of planar density, vertical height, and three-dimensional volume, respectively. While this functional association results in a high degree of numerical correlation, it does not alter the independent definitions and calculation logics of each indicator. The strong correlations among BD, MBH, and FAR demonstrate their mutual complementarity, collectively forming a comprehensive characterization of urban building morphology.

### 4.2. Characteristics of PM2.5 concentrations

#### 4.2.1. Spatiotemporal distribution of PM2.5.

**Fig 5:**
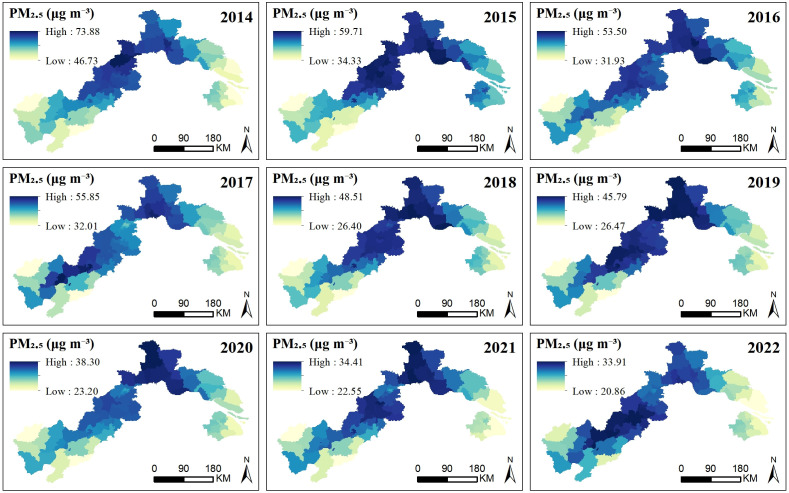
Spatial distribution of PM2.5 concentrations in the Lower reaches of the Yangtze River Delta, 2014–2022.

Overall, the PM2.5 concentration exhibits a spatial pattern of “high in the middle and low on both sides,”s with a significant temporal trend and stable spatial differentiation characteristics during the 2014–2022 period (see **Fig 5**).

**Fig 6:**
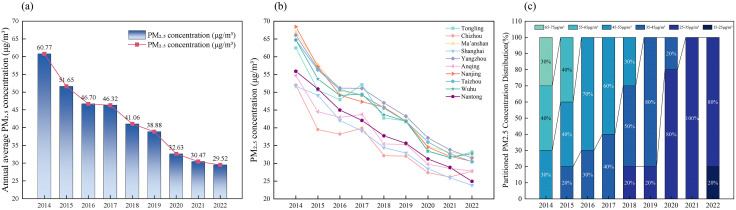
(a) Interannual variation of annual average PM2.5 concentration in the lower reaches of the Yangtze River Delta, 2014–2022; (b) Interannual variation of annual average PM2.5 concentration in various cities in the lower reaches of the Yangtze River Delta, 2014–2022; (c) Proportion of PM2.5 concentration intervals in the lower reaches of the Yangtze River Delta, 2014–2022.

From a temporal perspective, the annual average PM2.5 concentration across the lower reaches of the Yangtze River in the Yangtze River Delta has continuously decreased and stabilized (from 60.77 μg/m^3^ in 2014 to 29.52 μg/m^3^ in 2022). Among these, the decrease from 2016 to 2017 was the smallest, dropping from 46.70 μg/m^3^ to 46.32 μg/m^3^, a reduction of only 0.81%. In the past three years, the annual average PM2.5 concentration has stabilized, with declines ranging from 3.12% to 6.62%. PM2.5 concentrations in the cities of the downstream Yangtze River Delta continued to show a downward trend, with reductions as follows: Tongling 29.55%, Chizhou 32.68%, Ma’anshan 40.61%, Shanghai 29.58%, Yangzhou 29.87%, Anqing 30.34%, Nanjing 33.93%, Taizhou 30.03%, Wuhu 31.22%, and Nantong 29.97%. In terms of the overall concentration distribution, the proportion of high-concentration areas gradually decreased, while the proportion of low-concentration areas gradually increased. In 2014, areas with concentrations of 65–75 μg/m^3^ accounted for 30%, areas with 55–65 μg/m^3^ accounted for 40.00%, and areas with 45–55 μg/m^3^ accounted for 30.00%. By 2022, all concentration levels had shifted to 25–35 μg/m^3^ (accounting for 80.00% of the area) and 15–25 μg/m^3^ (accounting for 20.00% of the area) (see **Fig 6**).

**Fig 7:**
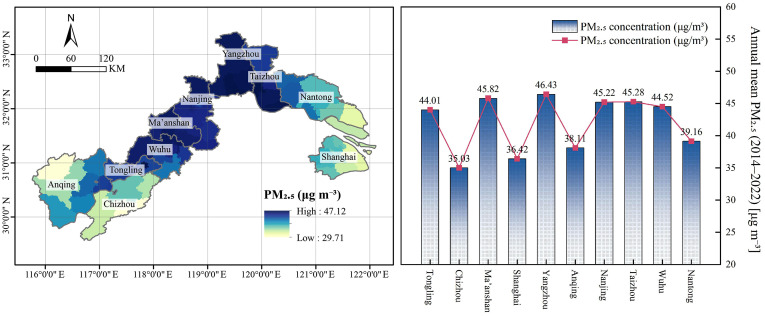
Spatial distribution of average PM2.5 concentrations in cities of the lower Yangtze River Delta, 2014–2022.

Spatially, PM2.5 concentrations generally formed a pattern centered on Nanjing and Ma’anshan, decreasing gradually outward. Multi-year average data showed that Yangzhou, Ma’anshan, Taizhou, and Nanjing had relatively high PM2.5 concentrations, at 46.43 μg/m^3^, 45.82 μg/m^3^, 45.28 μg/m^3^, and 45.22 μg/m^3^, respectively. In contrast, peripheral cities such as Chizhou, Shanghai, Anqing, and Nantong had lower concentrations, at 35.03 μg/m^3^, 36.42 μg/m^3^, 38.11 μg/m^3^, and 39.16 μg/m^3^, respectively. Spatial differences in PM2.5 concentrations were also observed within cities, particularly in peripheral cities (see **Table 3**). Within Anqing, eight levels of PM2.5 concentrations from low to high were observed, showing a spatial pattern of “low in the northwest and high in the southeast.” Similarly, Nantong exhibited significant variation in PM2.5 concentrations, with a pattern of “high in the northwest and low in the southeast.” In contrast, PM2.5 concentrations in cities such as Nanjing, Yangzhou, and Ma’anshan were predominantly concentrated in higher ranges, with a relatively uniform distribution (see **Fig 7**).

**Table 3:** Changes in PM2.5 concentration levels in the lower reaches of the Yangtze River Delta.

City	Province	PM2.5 concentration in 2014(μg/m^3^)	PM2.5 concentration in 2022(μg/m^3^)	Annual Average Change(μg/m^3^)	Mean PM2.5 Concentration(μg/m^3^)
Tongling	Anhui	62.4724	33.2109	−29.2615	44.0135
Chizhou	Anhui	52.0298	27.7853	−24.2445	35.0274
Ma’anshan	Anhui	66.9467	32.2375	−34.7092	45.8172
Shanghai	Shanghai	51.7202	23.8487	−27.8716	36.4196
Yangzhou	Jiangsu	66.0792	31.5111	−34.5682	46.4347
Anqing	Anhui	54.7065	27.8511	−26.8555	38.1094
Nanjing	Jiangsu	68.4441	30.5746	−37.8695	45.2239
Taizhou	Jiangsu	64.7086	30.4616	−34.2471	45.2772
Wuhu	Anhui	64.7249	32.7731	−31.9519	44.5151
Nantong	Jiangsu	55.9134	24.9304	−30.983	39.1563

#### 4.2.2. Spatial autocorrelation analysis of PM2.5 concentrations.

The results of the global Moran’s index for PM2.5 concentrations show that *Global Moran*’*s I* > 0, indicating spatial positive autocorrelation; *P* < 0.001, demonstrating strong significance; and *Z*-score > 2.58, confirming spatially clustered distribution characteristics. Specifically, both the *Moran’s I* and the *Z*-score exhibit considerable fluctuations. Notably, in 2014 and 2022, Global *Moran’s I* reached 1.106106 and 0.991845, respectively, reflecting strong spatial positive autocorrelation. Concurrently, the *Z*-scores also peaked during these years, indicating pronounced high-high spatial clustering (see **Table 4**).

**Table 4:** *Global Moran’s I* and test results of PM2.5 concentrations.

Year	*Global Moran’s I*	Z-value	P-value
2014	1.106106	15.825484	0.000000
2015	0.410307	6.058855	0.000000
2016	0.371409	5.510618	0.000000
2017	0.838768	12.058652	0.000000
2018	0.780104	11.231092	0.000000
2019	0.843034	12.126355	0.000000
2020	0.589076	8.558722	0.000000
2021	0.726923	10.485946	0.000000
2022	0.991845	14.207699	0.000000

**Fig 8:**
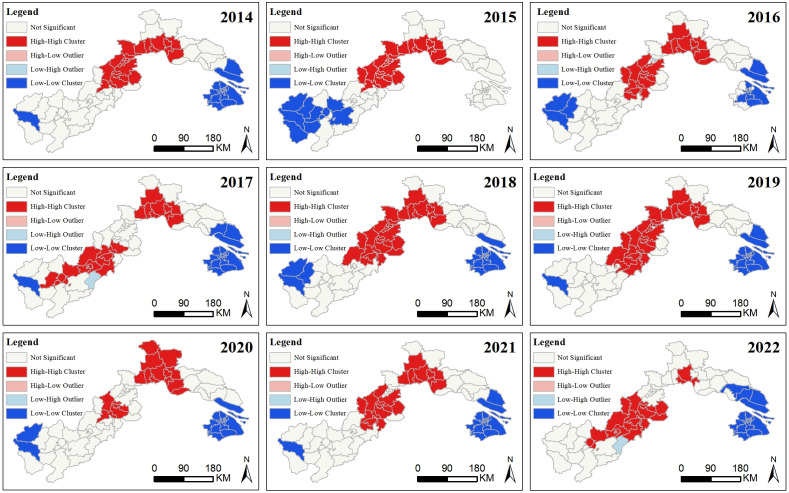
LISA distribution of PM2.5 concentration in the Lower Yangtze River Delta from 2014 to 2022.

The Local Moran’s I results indicate that the study area exhibits a spatial autocorrelation characteristic of high-high clusters in the central section and low-low clusters at the eastern and western ends. The spatial autocorrelation in the remaining areas is not significant, demonstrating an overall pronounced clustering pattern with high spatial consistency. Specifically, from 2014 to 2015, the majority of Nanjing, Yangzhou, Ma’anshan, and Taizhou showed high-high clustering. By 2017, Nanjing and its surrounding areas shifted to statistically insignificant associations, while parts of Wuhu and Tongling transitioned to high-high clustering. From 2018 to 2019, Nanjing reverted to a high-high clustering pattern, forming a contiguous distribution stretching from Wuhu to Taizhou from west to east. In 2020, the clustering pattern in Nanjing again became insignificant. Subsequently, high-high clustering areas expanded to the west of Nanjing while contracting on the east. The distribution of low-low clustering areas around Shanghai in the east remained relatively stable, whereas low-low clustering areas in the west exhibited greater fluctuations. In 2015, these expanded into urban areas of Anqing, Tongling, and Chizhou but continued to contract after 2020. By 2022, low-low clustering areas in the western region had disappeared (see **Fig 8**).

#### 4.2.3. Hotspot analysis.

The hot spot analysis (Getis-Ord Gi*) of PM2.5 concentrations reveals significant spatial differentiation between cold and hot spots in the study area. Hot spots are concentrated around Nanjing, a central city in the lower reaches of the Yangtze River within the Yangtze River Delta, while cold spots are clustered in peripheral cities such as Anqing and Shanghai. Furthermore, areas with different confidence levels exhibit a concentric circle distribution—areas with 95% and 90% confidence are predominantly distributed around areas with 99% confidence. Specifically, Nanjing, Ma’anshan, and Yangzhou are dominated by hot spots at the 99% confidence level, whereas Shanghai, Anqing, and Chizhou are dominated by cold spots at the 99% confidence level.

The spatiotemporal evolution characteristics of the hot spot areas are pronounced. From 2014 to 2015, hot spots were concentrated in Nanjing, southern Yangzhou, and Taizhou. Subsequently, the confidence level in Nanjing and its surroundings decreased, while it increased in southern Yangzhou and Taizhou. By 2017, Nanjing transitioned into a statistically insignificant area. Meanwhile, southern Yangzhou and Taizhou developed into hot spots. In 2018, the confidence level in Nanjing rose, and both it and the eastern regions of southern Yangzhou and Taizhou were identified as hot spots. Thereafter, the confidence levels in both Nanjing and Yangzhou declined. However, in 2020, the confidence levels in Yangzhou and Taizhou resumed an upward trend, while Nanjing reverted to a statistically insignificant area, and hot spots in Yangzhou and Taizhou expanded to their central-northern regions. In 2021, confidence levels increased in the area from southern Nanjing to Ma’anshan. By 2022, confidence levels rose in Wuhu and Ma’anshan, forming the only 99.00% confidence-level hot spot cluster, whereas confidence levels in Yangzhou and Taizhou decreased, trending toward statistical insignificance.

**Fig 9:**
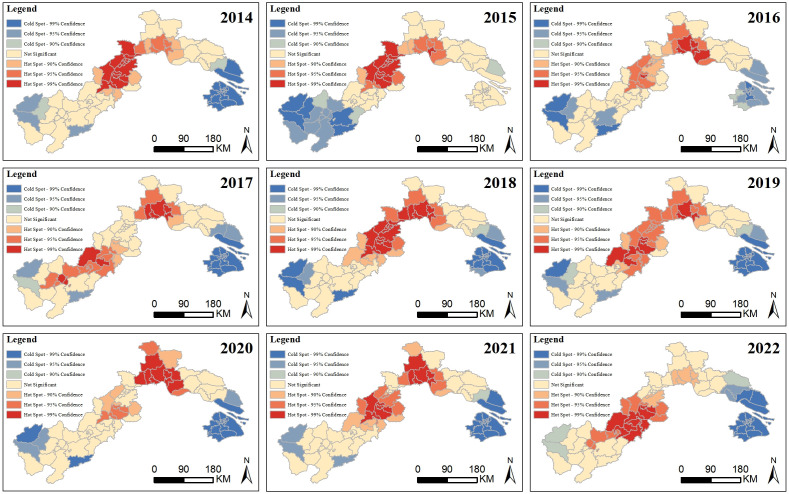
Spatial Characteristics of PM2.5 Concentration.

The cold spot areas exhibit an evolution pattern characterized as “stable in the east and dynamic in the west.”Cold spot areas around Shanghai in the east were relatively stable, mostly at the 99.00% confidence level, except in 2016 when a large area experienced a decline to a 95.00% confidence level. Cold spot areas in the western region exhibited greater fluctuations: significance increased from 2014 to 2015, forming the largest cold spot cluster by area in 2015. Confidence levels decreased from 2016 to 2017, with eastern Anqing and western Chizhou transitioning into insignificant areas. From 2018 to 2022, confidence levels for cold spots in the west showed a declining trend, and by 2022, only western Anqing retained a cold spot area with a 90.00% confidence level (see **Fig 9**).

### 4.3. Global driving mechanisms of PM2.5 concentrations based on OPGD

#### 4.3.1. Optimal discretization selection.

**Fig 10:**
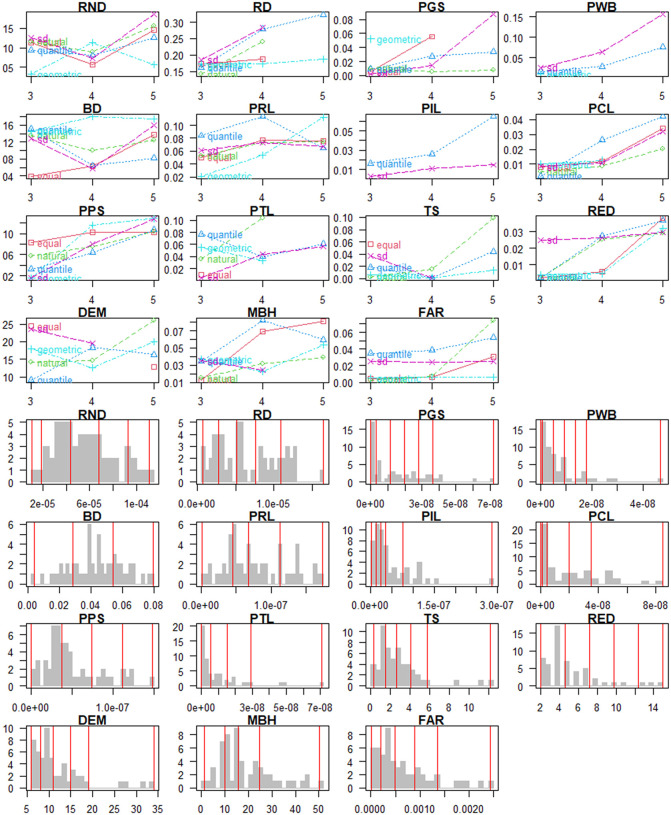
Discretization results.

Under the quantile classification method, the *q*-values for RD, PIL, PRL, PCL, and MBH were significantly superior to those obtained with other methods. When the optimal number of discretization bins was 4, the *q*-values for PRL and MBH reached their maxima (the discretization in the OPGD is primarily guided by optimizing the *q*-value, with the number of breakpoints flexibly adjusted according to data distribution characteristics). Specifically, all breakpoint values for PRL were less than 1, while the breakpoints for MBH were 1.58, 10.00, 15.87, 24.96, and 50.53, respectively. When the optimal number of discretization bins was 5, the q-values for RD, PIL, and PCL reached their maxima, with all corresponding breakpoint values less than 1 (see **Fig 10**).

In summary, based on the principle of maximizing the *q*-value, the number of discretization bins for BD, PRL, MBH, and BTH was set to 4, while that for the remaining factors was set to 5. The stratification method for RND, RD, PGS, PIL, PRL, PCL, and MBH was the quantile method; for RND, PGS, and PWB, the standard deviation (sd) method was selected; for BD, DEM, and FAR, the natural breaks method was used; and for TS and RED, the equal interval method was applied.

#### 4.3.2. Results of factor detection and interaction detection.

Factor detection results indicate that RD, DEM, and RND are significant factors influencing PM2.5 concentration. Their explanatory power (*q*-values) for PM2.5 spatial differentiation is 0.3232, 0.2604, and 0.1852, respectively. In contrast, the explanatory power of RED, PCL, and PIL is relatively weak, with *q*-values of 0.0382, 0.0422, and 0.0648, respectively.

**Fig 11:**
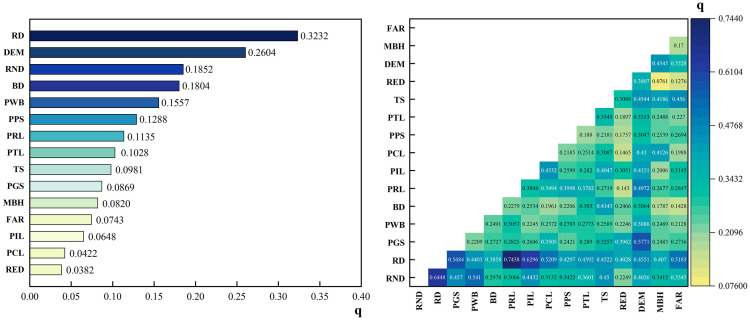
Single factor detection and interaction detection.

Interaction detection results show that the interactions between all factor pairs exhibited an enhanced explanatory power, with nonlinear enhancement being the predominant type. RD played a core role in the synergistic effects of multiple factors. Specifically, the interaction between PRL and RD exhibited the strongest explanatory power (*q* = 0.7438), followed by PIL × RD (*q* = 0.6296) and RED × RD (*q* = 0.4028). In the case of PRL × RD (*q* = 0.7438), PRL, by determining the density of residential land and the intensity of domestic emissions, acts as a “source” element for PM2.5; RD, by regulating local airflow and moisture conditions, functions as a “sink” for pollutants. The “source-sink” synergy between the two creates a nonlinear enhancement effect, with its explanatory power significantly exceeding the independent effects of single factors. For RED × RD (*q* = 0.4028), RED determines the fundamental influence of topography on airflow, while RD further optimizes or weakens ventilation conditions through river network corridors, synergistically affecting pollutant retention and transport. In the case of PCL × RD (*q* = 0.5209), emissions from mobile and domestic sources generated by commercial land use are dispersed and deposited through improved ventilation and moisture adsorption in waterfront areas, reflecting the combined effect of human activities and natural hydrological systems. (see **Fig 11**).

The results of the multi-scale comparative analysis show that the fluctuation range of the *q*-values of the core interaction pairs across different scales is less than 8%, and the interaction types consistently remain nonlinear enhancement, indicating that the interaction detection results of this study exhibit good spatial stability. Among the scales, the interaction explanatory power is optimal at the 20 km × 20 km scale, as it effectively avoids biases caused by data sparsity at smaller scales while preserving sufficient spatial heterogeneity. Therefore, this scale was determined as the final analysis scale (See **Table 5**).

**Table 5:** Multi-scale comparative analysis of interaction detection.

Spatial unit scale *q* value	PRL × RD	PIL × RD	RED × RD	Average fluctuation magnitude	Interaction type
10 km × 10 km	0.712	0.598	0.381	7.2%	nonlinear enhancement
20 km × 20 km	0.7438	0.6296	0.4028	–	nonlinear enhancement
30 km × 30 km	0.726	0.615	0.394	3.5%	nonlinear enhancement

#### 4.3.3. Results of risk detection and ecological detection.

**Fig 12:**
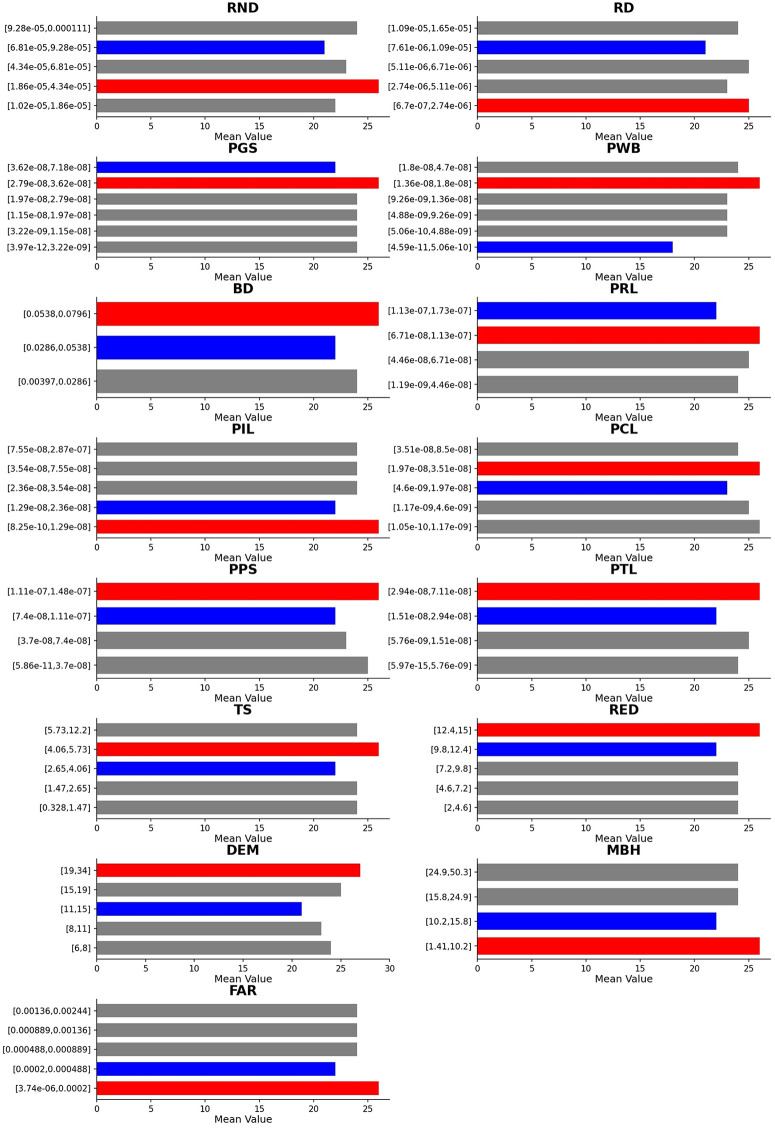
Mean values of categorized factors in risk detection.

Risk detection results indicate that the contributions of urban form factors from different dimensions and their numerical intervals to PM2.5 pollution risk vary significantly. Specifically: Within the 1D urban form, both RND and RD exhibit considerable influence. In areas where RD values fall within the range (6.7 × 10^−7^, 2.74 × 10^−6^], the PM2.5 concentration is 28.52 μg/m^3^, whereas in areas within the range (7.61 × 10^−6^, 1.09 × 10^−5^], the concentration decreases to 24.63 μg/m^3^. The pollution risk associated with RND is slightly lower; within its range of (1.86 × 10^−6^, 4.34 × 10^−5^], PM2.5 concentration reaches its highest level at 27.13 μg/m^3^. This reflects the phenomenon that areas with lower RD values tend to have higher pollution risks. Among 2D urban form indicators, the PWB and the PTL show significant impacts on PM2.5 pollution risk. In areas where PWB values are within (1.36 × 10^−8^, 1.8 × 10^−8^], the PM2.5 concentration reaches 29.82 μg/m^3^. When PTL values lie within (2.94 × 10^−8^, 7.11 × 10^−8^], the PM2.5 concentration is 28.97 μg/m^3^. For 3D urban form indicators, TS and DEM contribute notably to PM2.5 pollution risk. When TS values are within (4.06, 5.73], the PM2.5 concentration is 28.76 μg/m^3^. DEM demonstrates the highest pollution risk within the elevation range (19, 34], where PM2.5 concentration peaks at 30.06 μg/m^3^. Moreover, significant differences in PM2.5 concentrations are observed between areas with high and low values of RND, RD, PGS, RED, and DEM (see **Fig 12**).

**Fig 13:**
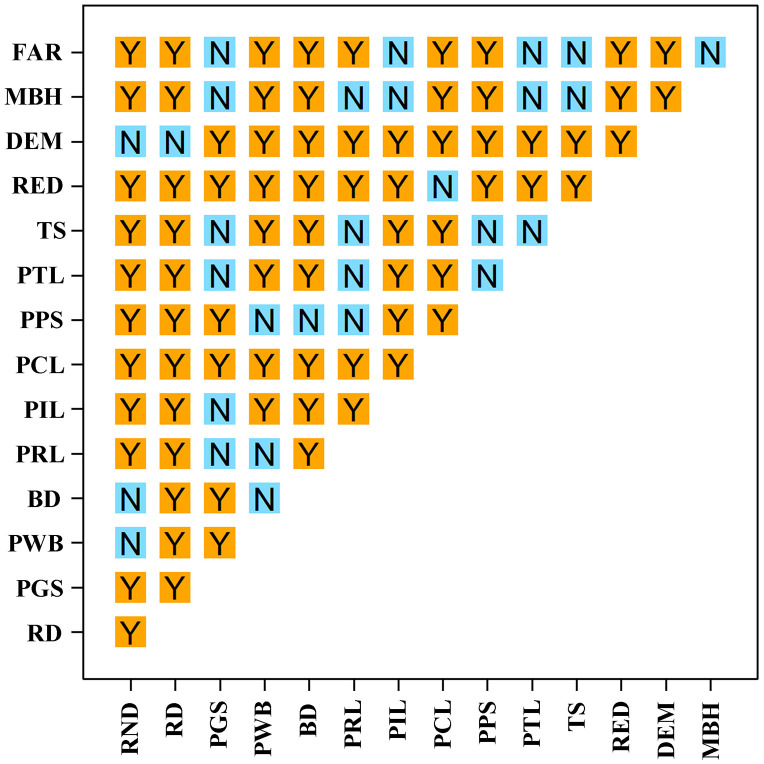
Ecological detection results.

Ecological detection results reveal that the impacts of most urban form factors on the spatial distribution of PM2.5 are significantly different from one another, with particularly pronounced differences observed among factors across different dimensions. Specifically: Significant disparities exist between factors across different dimensions. For example, RND (1D) differs markedly from PWB (2D), BD (2D), and DEM (3D) in their impacts on the spatial distribution of PM2.5 concentrations. A similar trend is observed between RD (1D) and DEM (3D). The influence of PGS (2D) differs significantly from that of multiple other factors, such as PRL (2D), PIL (2D), PTL (2D), TS (3D), MBH (3D), and FAR (3D). Additionally, significant differences persist between several 2D form factors and factors from other dimensions (e.g., PWB–BD, PRL, PPS; BD–PPS; PRL–PPS, PTL, TS, MBH; PIL–MBH, FAR; PCL–RED; PPS–PTL, TS; PTL–FAR, MBH, TS). Within the 3D forms, the influences of TS on PM2.5 concentration are significantly different from those of FAR and MBH, and the difference in influence between MBH and FAR is also relatively pronounced (see **Fig 13**).

#### 4.3.4. Results of sensitivity analysis for discretization schemes.

To verify the robustness of the *q*-values of the core driving factors (RD, DEM, RND) with respect to the discretization scheme and the number of bins, this study designed two sets of sensitivity tests. In each set, either the discretization method or the number of bins was held constant while only the other parameter was varied, to explore the ranking of the core factors and the magnitude of fluctuation in their *q*-values.

In both sets of tests, the fluctuation magnitude of the q-values for the core factors was less than 14% (with the maximum fluctuation being 13.82% for RND). The *q*-values of RD, DEM, and RND consistently ranked among the top three among all variables, with a stable order of RD > DEM > RND. No situation occurred where the core status was interchanged due to parameter adjustments. This indicates that although the explanatory power of the core factors is influenced by the discretization scheme or the number of bins, their overall stability is relatively strong. The discretization scheme of “optimal discretization method with 5 bins” enabled the *q*-values of the core factors to reach their peak (or near-peak) values, further demonstrating the scientific validity of the selected optimal discretization parameters (See **Table 6**, **Table 7**).

**Table 6:** Comparison of *q*-values of core driving factors under different discretization methods.

Core factor	Optimal method	Quantile	Standard deviation	Natural breaks	Equal interval	Geometric interval	Range of *q*-value fluctuation	Fluctuation magnitude
RD	0.3232	0.3232	0.2987	0.3051	0.2893	0.2946	0.0339	10.49%
DEM	0.2604	0.2418	0.2356	0.2604	0.2289	0.2327	0.0315	12.10%
RND	0.1852	0.1684	0.1852	0.1723	0.1596	0.1638	0.0256	13.82%

**Table 7:** Comparison of *q*-values of core driving factors under different numbers of bins.

Core factor	Optimal discretization bins	3 Bins	4 Bins	5Bins	6 Bins	7 Bins	Range of *q*-value fluctuation	Fluctuation magnitude
RD	0.3232	0.2910	0.3124	0.3232	0.3187	0.3153	0.0322	9.96%
DEM	0.2604	0.2350	0.2512	0.2604	0.2568	0.2531	0.0254	9.75%
RND	0.1852	0.1620	0.1743	0.1852	0.1815	0.1789	0.0232	12.53%

### 4.4. Local driving mechanisms of PM2.5 concentrations based on GW-XGBoost

#### 4.4.1. Ordinary least squares regression results.

To screen core driving variables and establish a global benchmark, an OLS regression analysis was first conducted on 15 multi-dimensional urban form indicators. Through a four-step screening process—significance testing, multicollinearity elimination, cross-validation with OPGD results, and model optimization—five core explanatory variables were ultimately identified. This ensured the use of key driving factors while avoiding redundancy in the subsequent GW-XGBoost model analysis.

The results of the full-indicator OLS model showed an adjusted coefficient of determination (Adjusted R^2^) of 0.736, indicating that the 15 indicators collectively explained 73.6% of the variation in PM2.5 concentrations. The overall model fit was significant (F-statistic = 38.62, P < 0.001). However, the model’s Akaike Information Criterion (AIC) value was as high as 124.3 (see **Table 8**), and mild multicollinearity (VIF > 1.5) was observed among BD (VIF = 2.147), MBH (VIF = 2.035), and FAR (VIF = 1.986), suggesting the presence of redundant variables (see **Table 9**).

**Table 8:** Goodness-of-fit and multicollinearity diagnostics for the all indicators.

Adjusted R²	AIC	Mean VIF	Number of variables
0.736	124.3	1.68	15

**Table 9:** Results of the all-indicator OLS regression analysis.

Category	Variable	Coefficient	Std.Error	t-value	P-value	VIF	Significance Level
	Constant	23.854	2.017	11.826	0.000	–	***
1D	RD	0.819	0.162	5.056	0.000	1.321	***
	RND	0.463	0.170	2.724	0.007	1.298	**
2D	PGS	−0.187	0.143	−1.308	0.192	1.684	–
	PWB	−0.315	0.137	−2.300	0.022	1.362	*
	BD	0.194	0.151	1.285	0.200	2.147	–
	PRL	0.126	0.118	1.068	0.286	1.873	–
	PIL	0.089	0.105	0.848	0.397	1.725	–
	PCL	0.074	0.096	0.771	0.441	1.598	–
	PPS	0.103	0.112	0.919	0.359	1.642	–
	PTL	0.281	0.129	2.178	0.030	1.279	*
3D	TS	0.156	0.114	1.369	0.172	1.431	–
	RED	0.062	0.089	0.697	0.486	1.385	–
	DEM	0.627	0.193	3.249	0.001	1.429	**
	MBH	0.098	0.087	1.126	0.261	2.035	–
	FAR	0.083	0.079	1.051	0.294	1.986	–

Based on four core principles—significance threshold, multicollinearity elimination, cross-validation with OPGD, and multi-dimensional form coverage—a final set of five variables was selected: RD, RND, PWB, PTL, and DEM. The P-values for RD (0.000), RND (0.007), DEM (0.001), PWB (0.022), and PTL (0.030) were all below 0.05, indicating high statistical significance. In contrast, the remaining ten variables (e.g., PGS, BD, PRL) exhibited P-values greater than 0.10, suggesting no significant global impact on PM2.5 concentrations, and were therefore excluded. The VIF for each of the five retained variables was below 1.50, fully satisfying the regression model assumptions regarding multicollinearity.

The selected variables align closely with the high-explanatory-power factors identified through OPGD factor detection. RD (*q* = 0.3232), DEM (*q* = 0.2604), and RND (*q* = 0.1852) ranked as the top three core factors in the OPGD results, while PWB and PTL demonstrated significant pollution risk contributions in OPGD risk detection. Furthermore, these five variables comprehensively cover the 1D, 2D, and 3D form dimensions: RD and RND represent 1D form; PWB and PTL represent 2D form; and DEM represents 3D form.

The Adjusted R^2^ of the final screened model is 0.728, only 0.008 lower than that of the full-indicator model. However, the AIC decreased significantly to 116.70 (a reduction of 7.60). The Durbin–Watson statistic of 1.95 indicates no autocorrelation, and the F-statistic reached 45.39 (P = 0.000), demonstrating a substantial improvement in model parsimony and fitting efficiency (see **Table 10**).

**Table 10:** Goodness-of-fit and multicollinearity diagnostics for the five core indicators.

Adjusted R²	AIC	Mean VIF	Number of variables
0.728	116.70	1.33	5

The global influence characteristics are clear: RD (0.832), DEM (0.631), RND (0.467), and PTL (0.285) exhibit significant positive effects on PM2.5 concentrations, whereas PWB (−0.318) shows a significant negative effect. These findings are fully consistent with both the OPGD risk detection outcomes and the underlying theoretical mechanisms, providing a robust global benchmark for the subsequent GW-XGBoost analysis of local spatial heterogeneity (see **Table 11**).

**Table 11:** Results of the final OLS regression analysis.

Category	Variable	Coefficient	Std.Error	t-value	P-value	VIF	Significance Level
**1D**	RD	0.832	0.161	5.168	0.000	1.319	***
	RND	0.467	0.171	2.731	0.007	1.296	**
**2D**	PWB	−0.318	0.136	−2.340	0.020	1.358	*
	PTL	0.285	0.128	2.227	0.027	1.275	*
**3D**	DEM	0.631	0.190	3.321	0.001	1.427	**
	Constant	24.219	1.896	12.774	0.000	–	***

#### 4.4.2. GW‑XGBoost results.

The model overfitting test results show: the training set achieved an R^2^ of 0.752 and an RMSE of 2.86 μg/m^3^, while the validation set achieved an R^2^ of 0.721 and an RMSE of 3.12 μg/m^3^, indicating a relatively small performance difference between the two. Model performance stabilized when the regularization parameter lambda was set to 0.5. The global *Moran’s I* of the residuals was 0.08 (*P* = 0.12 > 0.05), indicating no significant spatial autocorrelation. These results demonstrate that the GW-XGBoost model does not suffer from overfitting and exhibits good fitting accuracy and spatial generalization capability.

**Fig 14:**
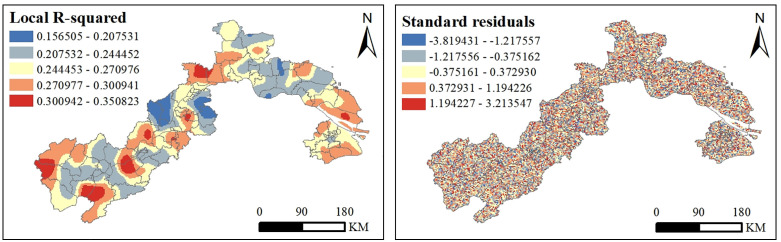
Spatial distribution of local R^2^ and standardized residuals.

The GW-XGBoost model was constructed using the five core variables (RD, RND, DEM, PWB, PTL) selected through OLS screening. The local R^2^ values of the GW-XGBoost model ranged from 0.16 to 0.35, exhibiting significant spatial heterogeneity. High local R^2^ values (0.30–0.35) were concentrated in highly urbanized areas such as Nanjing and Shanghai, where human activities are intensive and the regulatory effect of urban form on PM2.5 concentration is more prominent, resulting in stronger model explanatory power. Low local R^2^ values (0.16–0.20) were distributed in peripheral low-urbanization areas such as Anqing and Chizhou, as well as in most parts of Nantong. This indicates that PM2.5 concentrations in these areas are significantly influenced by external factors such as regional pollutant transport and meteorological conditions, in addition to urban form, leading to relatively limited local explanatory power of the model. The spatial distribution characteristics of the standardized residuals further validated the model’s fitting quality and unbiasedness from a spatial perspective. The standardized residuals were calculated for all grid cells and spatially visualized. The results show that the absolute values of the standardized residuals were generally less than 3, conforming to the criterion of a normal distribution. Only a few scattered high-residual cells (standardized residual > 3) were observed in the eastern coastal area of Nantong and the western mountainous area of Anqing. Furthermore, both high and low residuals exhibited a random, discrete spatial distribution without forming continuous clusters. This finding demonstrates that the GW-XGBoost model, through its geographically weighted and nonlinear fitting mechanisms, effectively reduces systematic fitting bias caused by uneven spatial distribution, thereby establishing a reliable model foundation for subsequent testing of whether variable relationships exhibit spatial non-stationarity. (see **Fig 14**).

**Fig 15:**
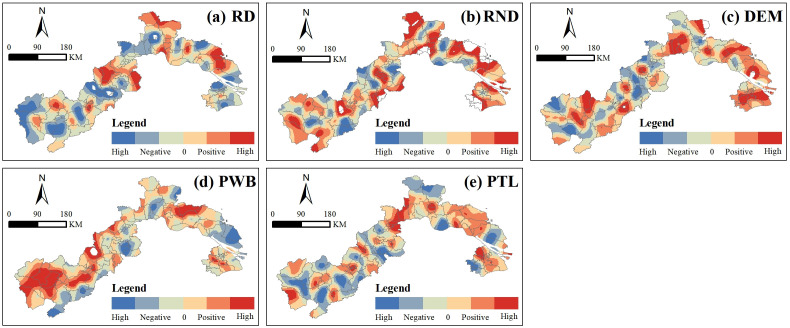
Spatial distribution of local regression coefficients of the core variables (RD, RND, DEM, PWB, PTL) in the GW-XGBoost model.

The local regression coefficients of the five core variables exhibited distinct regional differentiation patterns in their spatial distribution, exerting differentiated impacts on PM2.5 concentration. This reflects the spatial heterogeneity in the regression relationships between PM2.5 concentration and the core variables. The local regression coefficients of RD showed a positive driving effect in most regions, with the dark red areas (coefficient values ranging from 0.80 to 1.00) concentrated in the central part of the study area, specifically the Nanjing–Ma’anshan–Wuhu belt. In contrast, the proportion of blue areas increases in open regions with sparse river networks, such as Anqing and Chizhou, where coefficients drop to 0.60–0.80, indicating a significantly weakened positive driving effect. The local regression coefficient for RND shows pronounced spatial variation: deep red areas (coefficients 0.50–0.70) appear in transportation hubs, such as the main urban area of Nanjing and northern Shanghai. In suburban and peripheral counties, blue areas expand, with coefficients approaching zero. The spatial pattern of the local regression coefficient for DEM varies: concentrated red areas (coefficients 0.70–0.90) are observed in the 0–30 m elevation zones near mining areas in Tongling and steel plants in Ma’anshan. In higher-altitude areas (86.00–255.00 m), light red or blue dominates, indicating a relatively moderate positive driving effect. The coefficient for PWB shows significant blue values (coefficients –0.40 to –0.30) along the Yangtze River and around large lakes, reflecting a strong mitigating effect of water bodies on PM2.5. In inland areas distant from water bodies, the proportion of red increases, and the absolute value of the negative coefficient decreases to 0.10–0.20, indicating a substantially weakened mitigating effect. For PTL, deep red areas (coefficients 0.30–0.50) appear along expressways and near logistics hubs. In areas dominated by residential and ecological functions, light red and blue prevail, with coefficients dropping to 0.10–0.20, suggesting a relatively mild driving effect (see **Fig 15**).

## 5. Discussion

### 5.1. Characteristics of PM2.5 concentrations

The PM2.5 concentrations form a hotspot area centered on Nanjing and Ma’anshan, gradually decreasing toward the east and west. This pattern is consistent with the findings of [32]. The elevated concentrations in Nanjing result from the combined effects of industrial production and residential emissions in the main urban area, exacerbated by the unfavorable topography of being surrounded by mountains on three sides, which hinders pollutant dispersion [54]. Ma’anshan, primarily a heavy industrial hub—especially for steel production—emits substantial pollutants, likely contributing to persistently high PM2.5 levels in the region. The annual average PM2.5 concentrations in the downstream Yangtze River Delta show a declining trend, with Ma’anshan experiencing the largest reduction (40.61%). Notably, Nanjing’s PM2.5 concentration dropped sharply in 2017, forming a low-value center. Since the implementation of the “Blue sky defense campaign”, Ma’anshan has adopted precise pollution control measures targeting coal, industrial gases, vehicles, dust, and open burning, utilizing modern monitoring methods to identify and address air pollution sources and actively promoting pollution control projects. While these efforts have yielded significant results, PM2.5 concentrations remain relatively high and have not been fundamentally controlled [55]. This outcome reflects the impact of the Air Pollution Prevention and Control Action Plan (APPCAP) introduced in 2013 and the implementation of related pollution control measures in Nanjing [56], leading to the low PM2.5 concentration observed in 2017. Shanghai and Anqing form cold spots with lower PM2.5 concentrations. Relevant studies indicate that air pollutants in Shanghai are significantly influenced by cross-regional atmospheric transport. The multi-regional collaborative air pollution control strategies adopted in Shanghai, such as establishing a joint air pollution early-warning center for sharing monitoring data across the Yangtze River Delta and temporarily shutting down polluting factories during events like the G20 Summit in Hangzhou, have effectively mitigated PM2.5 pollution [57]. Anqing, characterized by flat terrain located between the Dabie Mountains and the Huangshan remnants, acts as a corridor for PM2.5 transport, reducing pollutant retention [58].

### 5.2. Characteristics of the global driving mechanisms of PM2.5

This study innovatively constructs an analytical framework for a “multi‑dimensional urban form system” and integrates spatial analysis techniques with the OPGD method. This approach accurately reveals the complex, spatially heterogeneous, and nonlinear driving mechanisms between PM2.5 concentrations and urban form factors in the Yangtze River Delta region, thereby addressing the limitations of traditional research. RD, DEM, and RND are the most influential factors for PM2.5 concentrations, with explanatory powers (*q*-values) of 0.3232, 0.2604, and 0.1852, respectively. Water bodies can promote the hygroscopic settlement of particulate matter and enhance air flow through local cooling effects, thereby facilitating the dispersion of PM2.5 [59]. Related studies indicate that road transportation is a significant source of air pollution, with CO_2_ emissions from vehicles showing a positive correlation with PM2.5 concentrations [60]. Topography has also been confirmed as a critical factor influencing PM2.5 [61]. The downstream Yangtze River Delta region, characterized by mountains and hills, forms unique landforms such as valley plains and low-lying basins, which affect atmospheric circulation and hinder pollutant dispersion [62]. For instance, the valley topography of Nanjing, surrounded by mountains on three sides, is one of the key reasons for its persistently high PM2.5 levels. Additionally, low-elevation areas often exhibit high PM2.5 emissions due to intensive human activities [63], such as steel plants in Ma’anshan, which exacerbate gaseous PM2.5 pollution. In contrast, RED, PCL, and PIL show lower explanatory power for PM2.5 concentrations. However, under two-factor interactions, their interactive explanatory powers with RD increase to 0.4028, 0.5209, and 0.6296, respectively. The downstream Yangtze River Delta region consists mainly of plains and hills with relatively small overall topographic relief; thus, the blocking effect of high mountains on PM2.5 is not prominent [64]. In practice, differences in PM2.5 concentration distributions are primarily driven by local micro-topographic variations, resulting in the weak explanatory power of RED. The enhanced regional connectivity and resource allocation efficiency in downstream cities, along with the integrated development of sub-centers and core metropolises, have optimized industrial structures and promoted green economies [65]. This has increased the share of commercial and service sectors, diluting the dominance of single industrial structures. Leveraging synergistic development advantages, production technologies in surrounding cities have also improved [66]. The optimization of industrial structures and production technologies has reduced PM2.5 emissions at the source, which explains the limited explanatory power of PCL and PIL for increased PM2.5 concentrations. The interactive effect of RD lies in the blocking role of water bodies on PM2.5. Water bodies can transport PM2.5 emitted from industrial and commercial activities on surrounding plains to areas directly above the water via lake-breeze circulation, thereby hindering the diffusion of PM2.5 to downwind urban areas [67].

The PM2.5 pollution risk is highest (concentration reaching 30.06 μg/m^3^) within the DEM range of (19, 34] m. This indicates higher local PM2.5 concentrations at relatively higher elevations, which contrasts with the typical accumulation effect of PM2.5 in low-elevation basins. This phenomenon occurs because heavy-industry plants and related human activities tend to cluster in high-elevation mountainous or suburban areas to minimize impacts on urban residents or maximize resource utilization, as seen in the Tongling mining area [68] and Ma’anshan steel plants. RND and RD also substantially influence PM2.5 pollution risk. The highest PM2.5 concentration (28.52 μg/m^3^) occurs in areas with RD values in the range (6.7 × 10^−7^, 2.74 × 10^−6^]; when RND is in the range of (1.86e-06, 4.34e-05], the PM2.5 concentration reaches its highest value, at 27.13 μg/m^3^.This suggests that lower RND and RD values are associated with higher PM2.5 pollution risks. Lower RND often reflects an irrational road network structure, where vehicles concentrate on major roads, increasing congestion risks and promoting local PM2.5 generation [69].

### 5.3. Characteristics of local driving mchanisms of PM2.5

The results of the GW-XGBoost model reveal significant spatial non-stationarity in the relationship between PM2.5 concentrations and urban form factors. The local effect strength and direction of core driving variables exhibit distinct regional differentiation, which is closely coupled with urban development levels, topographic characteristics, and functional layouts.

In terms of spatial differentiation patterns, the driving mechanisms differ significantly between highly urbanized areas and peripheral, less-urbanized regions. Core cities such as Nanjing and Shanghai achieve Local R^2^ of 0.30–0.35, indicating a more pronounced regulatory role of urban form on PM2.5 concentrations. These areas feature dense populations and concentrated building and transportation networks. For example, the local regression coefficient of RND in the main urban area of Nanjing reaches 0.50–0.70, consistent with findings that PM2.5 concentrations are highest near traffic hubs [70], highlighting the significant cumulative effect of PM2.5 due to traffic congestion [71]. In contrast, peripheral areas such as Anqing and Chizhou show Local R^2^ values of only 0.16–0.20, and large parts of Nantong also exhibit low determination coefficients, suggesting a weaker influence of urban form. This may be attributed to the interference of external factors such as meteorological conditions and regional pollutant transport [72]. Influenced by northwesterly and northerly winds, PM2.5 in southern Anhui and Nantong primarily originates from cross-border transport from neighboring provinces or cities, which aligns with the findings of [73,74].

The positive driving effect of RD exhibits a “central agglomeration” characteristic. In the central region of Nanjing–Ma’anshan–Wuhu, the local regression coefficients of RD reach 0.80–1.00, reflecting the synergistic effect of dense river networks and high-intensity human activities [75]. The area above water bodies is prone to the accumulation of industrial pollutants and traffic emissions due to thermal circulation, while restricted ventilation in built-up areas exacerbates pollutant accumulation. This is consistent with the OPGD interaction detection results (PRL × RD *q*-value = 0.7438), verifying the synergistic influence mechanism of river networks and land use on PM2.5. The positive driving effects of RND and the PTL exhibit a “transportation-hub-focused” pattern. In transportation hubs such as the main urban area of Nanjing and northern Shanghai, the RND coefficient reaches 0.50–0.70. In freight-concentrated areas along expressways and near logistics hubs, the PTL coefficient ranges from 0.30 to 0.50, both showing deep-red clustering characteristics. These areas feature dense road networks and highly concentrated traffic flow, resulting in substantial vehicle exhaust emissions that are exacerbated by congestion, leading to increased pollutant accumulation [76]. The positive driving effect of the DEM is primarily concentrated in “specific elevation zones”. In the 0–30 m elevation zones around the Tongling mining area and Ma’anshan steel plants, as well as in lower-elevation regions such as Nanjing and Nantong, the DEM coefficient reaches 0.70–0.90. These elevation zones experience high anthropogenic emission intensities and are prone to temperature inversions that hinder vertical pollutant dispersion. The superposition of industrial emissions further intensifies the positive driving effect of DEM on PM2.5 concentrations. In contrast, higher-altitude mountainous areas benefit from frequent airflows and superior diffusion conditions [77]. The negative driving effect of the PWB follows a “proximity-to-water attenuation” pattern. Along the Yangtze River and around large lakes, the PWB coefficient ranges from –0.40 to –0.30, reflecting a strong mitigating effect on PM2.5 pollution. This is consistent with the conclusions of [78] and reflects the high ecosystem service value of water bodies [79]. Water bodies adsorb PM2.5 from the air through hygroscopic sedimentation, while lake-breeze circulation improves local ventilation conditions; these dual mechanisms collectively reduce PM2.5 concentrations [80]. In inland areas distant from water bodies, the absolute value of the negative coefficient decreases to 0.10–0.20, indicating the spatial limitation of water bodies in reducing pollution.

### 5.4. Strategies and recommendations

Based on the driving mechanisms of PM2.5 concentrations revealed by the OPGD and GW‑XGBoost models, combined with the urban form characteristics and spatial heterogeneity of the downstream Yangtze River Delta urban agglomeration, the following urban planning and pollution prevention and control optimization strategies are proposed:

(1) Precise regulation of core factors

Targeting the three core driving factors with the greatest impact on PM2.5—RD, DEM, and RND—urban form and spatial layout should be optimized. In high-risk areas with low RD values (e.g., certain industrial clusters), continuous river network systems should be established by excavating artificial wetlands and restoring natural waterways to leverage the hygroscopic deposition function of water bodies. In high-risk elevation zones of 19–34 m DEM (e.g., around the Tongling mining area and Ma’anshan steel plants), strict controls should be imposed on new industrial land use, promoting technological upgrades and relocation of high-pollution enterprises while improving regional ventilation conditions to mitigate the blocking effect of temperature inversions. To address congestion-related pollution caused by irrational RND configuration, secondary and branch road networks should be densified in areas with sparse road networks, forming a three-level “arterial–secondary–branch” road system to disperse traffic flow.

(2) Regionally differentiated management

Based on the spatial differentiation characteristics of local driving mechanisms, targeted control strategies should be implemented. For core cities (Nanjing, Shanghai), efforts should focus on the coordinated optimization of building morphology and the transportation system. This includes planning north-south ventilation corridors to preserve mountain ventilation gaps, while simultaneously optimizing the road network structure by densifying secondary and branch road networks to disperse traffic flow, thereby reducing emission concentrations in transportation hub areas and mitigating the synergistic pollution contribution of RND and construction land. In peripheral cities (e.g., Anqing and Chizhou), priority should be given to strengthening the construction of ecological buffer zones, using river networks and green spaces to create pollutant diffusion pathways and mitigate the impacts of regional transport. For areas with low local determination coefficients, such as Nantong, cross‑regional pollution prevention and control mechanisms should be established to manage both regional transport and local emission contributions, achieving coordinated governance.

(3) Synergistic enhancement of multi-dimensional form

Promote the coordinated optimization of 1D-2D-3D form indicators to enhance integrated pollution control effectiveness. Focus on the structural optimization and functional synergy of river and road networks, strengthen the construction of urban blue-green networks, and form an interwoven “water-green” pollutant interception and deposition network in coordination with green corridors at the 2D level. At the 2D form level, optimize the spatial layout of PGS and construction land by establishing wedge-shaped green corridors between industrial and residential zones to enhance pollutant interception capacity. At the 3D form level, control the density and height of high-rise buildings to avoid exacerbating heat island effects, preserve ecological green spaces in high-DEM areas, and rationally plan industrial layouts in low-lying plains. Through the coordinated regulation of multi-dimensional forms, a three-level pollution control system of “emission control–diffusion promotion–sedimentation enhancement” can be established.

### 5.5. Limitations and future perspectives

While this study systematically analyzed the influence mechanisms of multi-dimensional urban form on PM2.5 concentrations from both global and local perspectives by introducing the OPGD and GW-XGBoost methods, several limitations remain. First, both the OPGD and GW‑XGBoost models did not fully incorporate meteorological factors (e.g., wind speed, precipitation). Future research could expand data types to include multi-dimensional factors such as meteorology and industrial structure, thereby enriching the analytical dimensions of driving mechanisms and their interactions with urban form. Second, regarding the research scope, the study treated the downstream Yangtze River Delta urban agglomeration as a whole, without conducting differentiated mechanism analyses for cities of different scales and functional types (e.g., industrial cities, comprehensive cities). Future work could expand the study area to the entire Yangtze River Delta urban agglomeration and compare the driving mechanisms across cities with different functional types, providing a more comprehensive scientific basis for cross-regional and differentiated pollution prevention and control policy formulation.

## 6. Conclusion

This study, addressing the practical need for PM2.5 pollution prevention and control in the context of urbanization and population expansion, systematically analyzes the spatiotemporal evolution characteristics of PM2.5 concentration from 2014 to 2022. This study innovatively constructs a 1D-2D-3D multi-dimensional urban form analysis framework, and for the first time integrates the OPGD and GW-XGBoost models to systematically and accurately resolve the driving mechanisms of multi-dimensional urban form on PM2.5 concentrations in 2022 from both global and local dual perspectives. This addresses the shortcomings of existing studies in terms of systematicity and accuracy, including incomplete multi-dimensional urban form systems, inadequate characterization of nonlinear global driving effects, and insufficient analysis of spatially heterogeneous local processes. The main conclusions are as follows: (1) Regional PM2.5 concentration shows a decreasing trend with a stable spatial pattern (continuously decreasing from 60.77 μg/m^3^ to 29.52 μg/m^3^, with reductions ranging from 29.55% to 40.61% across individual cities). Spatially, it consistently maintains a stable pattern of “high in the middle and low on both sides,” forming a hot spot area at the 99% confidence level centered around Nanjing and Ma’anshan. (2) Multi-dimensional urban form exhibits significant spatial differentiation and prominent vertical expansion characteristics. High values of 1D forms (RND, RD) and 2D forms (PGS, BD, PRL, etc.) are concentrated east of Nanjing, while high values of 3D forms (TS, RED, DEM) are distributed west of Nanjing. BD shows a strong positive correlation with MBH and FAR, reflecting the trend of urban vertical expansion. (3) The global driving mechanism is characterized by the dominance of core factors and interaction enhancement. RD, DEM, and RND are the three core factors influencing PM2.5 concentration, with q-values of 0.3232, 0.2604, and 0.1852, respectively. All factor interactions exhibit nonlinear enhancement effects, indicating that the impact of urban form on PM2.5 concentration is a complex process involving the synergistic effect of multiple factors. Risk detection reveals that pollution risk is highest when DEM is in the 19–34 m elevation zone, with PM2.5 concentration reaching 30.06 μg/m^3^. (4) Significant spatial non-stationarity exists in the local driving mechanisms, which are highly coupled with urban development characteristics. In core cities, RD, RND, DEM, and PTL exhibit significant positive driving effects, while PWB shows a mitigating negative effect characterized by “water-adjacent attenuation.” In peripheral cities (Anqing, Chizhou), the local coefficients of determination (R^2^) are only 0.16–0.20, indicating more significant interference from regional transport and meteorological conditions. This study clarifies the global core driving factors and locally differentiated driving characteristics of multi-dimensional urban form on PM2.5 concentration in the lower reaches of the Yangtze River urban agglomeration within the Yangtze River Delta. It reveals the spatial heterogeneity in the influence of urban form on PM2.5 concentration, providing a specific scientific basis for precise PM2.5 pollution prevention and control and urban form optimization in this region. Furthermore, the multi-dimensional urban form analysis framework constructed and the integrated OPGD and GW-XGBoost research methods employed in this study can serve as methodological references for similar research in other high-density urban agglomerations. This holds significant practical value for promoting green urban development, improving atmospheric environmental quality, and achieving coordinated development between urbanization and the ecological environment.

## Supporting information

S1 File Data. (ZIP)
